# Convolutional Neural Network Intelligent Segmentation Algorithm-Based Magnetic Resonance Imaging in Diagnosis of Nasopharyngeal Carcinoma Foci

**DOI:** 10.1155/2021/2033806

**Published:** 2021-08-13

**Authors:** Deli Wang, Zheng Gong, Yanfen Zhang, Shouxi Wang

**Affiliations:** ^1^Department of Otorhinolaryngology, The Second Affiliated Hospital of Shandong First Medical University, Tai'an 271000, Shandong, China; ^2^Department of Otolaryngology, Heze Municipal Hospital, Heze 274031, Shandong, China

## Abstract

The aim of this study was to explore the adoption value of convolutional neural network- (CNN-) based magnetic resonance imaging (MRI) image intelligent segmentation model in the identification of nasopharyngeal carcinoma (NPC) lesions. The multisequence cross convolutional (MSCC) method was used in the complex convolutional network algorithm to establish the intelligent segmentation model two-dimensional (2D) ResUNet for the MRI image of the NPC lesion. Moreover, a multisequence multidimensional fusion segmentation model (MSCC-MDF) was further established. With 45 patients with NPC as the research objects, the Dice coefficient, Hausdorff distance (HD), and percentage of area difference (PAD) were calculated to evaluate the segmentation effect of MRI lesions. The results showed that the 2D-ResUNet model processed by MSCC had the largest Dice coefficient of 0.792 ± 0.045 for segmenting the tumor lesions of NPC, and it also had the smallest HD and PAD, which were 5.94 ± 0.41 mm and 15.96 ± 1.232%, respectively. When batch size = 5, the convergence curve was relatively gentle, and the convergence speed was the best. The largest Dice coefficient of MSCC-MDF model segmenting NPC tumor lesions was 0.896 ± 0.09, and its HD and PAD were the smallest, which were 5.07 ± 0.54 mm and 14.41 ± 1.33%, respectively. Its Dice coefficient was lower than other algorithms (*P* < 0.05), but HD and PAD were significantly higher than other algorithms (*P* < 0.05). To sum up, the MSCC-MDF model significantly improved the segmentation performance of MRI lesions in NPC patients, which provided a reference for the diagnosis of NPC.

## 1. Introduction

Nasopharyngeal carcinoma (NPC) is a common malignant tumor, and the incidence of NPC in China accounts for 80% of the world [1]. At present, X-ray, computed tomography (CT), positron emission computed tomography (PET), and magnetic resonance imaging (MRI) are often used to diagnose NPC patients. X-rays are limited to the morphological level, and it is difficult to make early and accurate positioning for problems such as recurrence [2]. CT is currently the main method for locating NPC lesions, but CT diagnosis mainly relies on the tumor's space-occupying effect on normal tissues, and it is difficult to find early small lesions without space-occupying effect on isodensity lesions and hidden parts [3]. The PET method obtains more accurate staging and improves the accuracy of target area delineation. However, there is currently no uniform clinical tumor target area delineation standard, and there are obvious differences in the target area visually delineated by different observers [4]. MRI examination is widely used in the diagnosis of various diseases because it is noninvasive, painless, and reproducible. The three sequences of T1W, T2W, and T1C in MRI images can clearly reflect the anatomical structure, tissue lesions, and microvessels, respectively. It is complementary in reflecting the structure of NPC tumors, lymph nodes, and surrounding tissues and organs and the occurrence of disease [5]. MRI images can show the shape, size, and location information of the lesion. At present, MRI diagnosis of NPC lesions mainly relies on the doctor's manual delineation, which is prone to missed diagnosis and misdiagnosis [6]. Due to the complexity and change of tumor location, shape, and size, blurring of the boundary between the tumor area and normal tissue, and uneven gray scale of the diseased tissue in the diagnosis of NPC, it is difficult to segment NPC tumor lesions [7]. Therefore, it is of great significance to find an automatic, accurate, and effective NPC tumor segmentation method to assist doctors in diagnosis.

Convolutional neural network (CNN) model integrates the automatic feature extraction and selection process in the data training process and solves the problems of local minimum or nonconvergence [8]. CNN is widely used in medical image segmentation, registration, classification, and so on due to its powerful fitting ability and the advantages of automatic feature extraction. Wang et al. [9] used deep de-CNN to segment NPC lesions, and the results showed that deep anti-CNN can effectively improve the segmentation effect of NPC tumors and lymph nodes compared with VGG network. Ye et al. [10] used CNN to segment the lesion area of the MR image of NPC patients and obtained an ideal segmentation effect. However, the segmentation method based on the CNN algorithm uses a single two-dimensional (2D) image to segment, and the information reflected by the 2D single-sequence image is limited, which ultimately affects the efficiency of image segmentation [11], and it needs to be further optimized.

In summary, the segmentation method based on the CNN algorithm needs to be further optimized. Based on CNN, a multisequence multidimensional fusion segmentation model (MSCC-MDF) was established and applied to the segmentation of NPC lesions, to provide a reference for the diagnosis of NPC.

## 2. Materials and Methods

### 2.1. Experimental Data

A total of 45 patients with NPC confirmed by histology in our hospital from December 2018 to December 2020 were selected as the research subjects. All patients underwent MRI examination. There were 32 males and 13 females, ranging in age from 18 to 78 years. The mean age was 40.84 ± 6.92 years. Inclusion criteria for this study were (i) patients diagnosed as nonkeratinized undifferentiated carcinoma by nasopharyngeal lesion biopsy; (ii) patients without any antitumor treatment; (iii) the clinical data and imaging data of the patients being complete; (iv) no primary tumor occurring in other sites. Exclusion criteria were (i) patients who were unsuitable for radiotherapy; (ii) patients with severe abnormal liver and kidney function or with other cancers; (iii) MRI image being not clear and not meeting the clinical adoption standards. The experimental procedure of this study had been approved by the Ethics Committee of the Hospital, and all the subjects included in the study had signed the informed consent.

### 2.2. MRI Examination Methods and Diagnostic Criteria

All study subjects were diagnosed with Siemens Verio3.0T MRI scanner, and all patients underwent MRI plain scan, enhanced scan, and DWI scan. Axial and coronal rapid small-angle excitation 2D sequence T1WI scanning parameters were as follows. The repetition time (TR) was 250 ms, the echo time (TE) was 250 ms, the scanning field (FOV) was 185 mm × 220 mm, and the matrix was 216 × 265. The T2WI scanning parameters of the fast spin echo sequence and fat suppression sequence were as follows: TR = 3900 ms, echo time TE = 92 ms, FOV was 189 mm × 30 mm, and matrix was 256 × 256. The T1C image scanning parameters were as follows: TR = 650 ms, TE = 9.17 ms, FOV = 200 mm, and layer thickness = 5 mm. The resolution of all MRI images was 512 × 512, and the tumor area and lymph nodes were delineated by two experienced clinicians.

MRI diagnostic criteria for NPC: NPC tumor tissue was equal or slightly high signal, and the surrounding tissue of the tumor was enhanced, but the surrounding tissue was not enhanced.

### 2.3. The Establishment of Intelligent Segmentation Model Based on CNN

The core convolutional layer of CNN plays the function of image feature extraction in the network [12]. The input size, convolution kernel size, step size, and filling method in the convolution operation will all have a certain impact on the output size [13]. The relationship between the output size of the convolution operation and various factors is expressed as follows:

(1) $$\begin{array}{c} O=\frac{I-C+2F}{S}+1, \end{array}$$

where *O* is the output size, *I* is the input size, *C* is the convolution kernel size, *F* is the padding size, and *S* is the step size.

Segmentation of the lesion area of the MRI image of NPC patients requires identification of the edge information of the NPC lesion and surrounding tissues. The maximum pooling operation is selected to reduce the model parameters to maintain the translation and rotation invariance of features in the feature extraction process [14]. The maximum pooling operation can retain the maximum value of the pooling window in MRI image feature extraction and reduce the deviation of the estimated mean value in the convolution process [15]. With the increase of network depth in CNN, gradient dispersion and gradient disappearance are prone to occur in the process of network operation, which makes network training difficult or even impossible to train. The residual structure in the ResNet network sums the input and the input transformed by the convolutional layer, which can significantly reduce the difficulty of training [3]. It is assumed that the network input of CNN is *A*, the expected output is *E*(*A*), and *A* is transformed into *F*(*A*) after multiple convolutional layer operations; then the relationship between *E*(*A*) and *F*(*A*) is expressed as follows:

(2) $$\begin{array}{c} E\left(A\right)=F\left(A\right)+A. \end{array}$$

Then the learning goal of this part of the network is expressed as follows:

(3) $$\begin{array}{c} F\left(A\right)=E\left(A\right)-A. \end{array}$$

**Figure 1:**
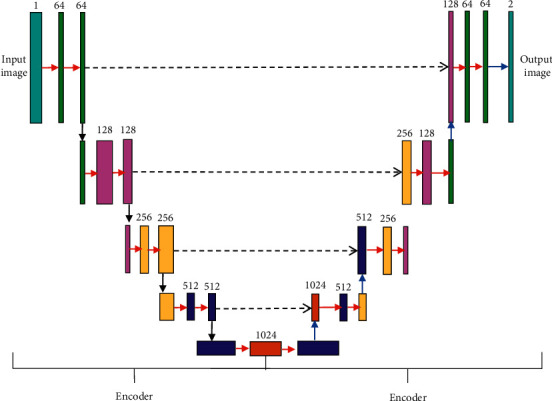
Encoding and decoding structure diagram based on CNN algorithm.

The encoding and decoding structures play an important role in image processing. Encoding refers to using convolutional layer and pooling layer to obtain image feature maps during MRI image processing. Decoding refers to using convolutional layers and transposed convolutional layers to convert feature maps into feature maps required for specific tasks. The encoding and decoding structure with skip connections based on the CNN algorithm can merge the feature maps of different resolutions obtained by the encoder into the feature maps of the corresponding size of the decoder. Then, convolution operation is performed on the obtained feature map. The encoding and decoding structure based on the CNN algorithm can ensure the repeated use of feature maps of the same size and reduce the loss of information in the pooling of the encoding stage. The encoding and decoding structure diagram based on the CNN algorithm is shown in Figure 1.

The activation function has an activating effect on neurons [16]. In this research, linear rectification function (ReLU) was utilized as the activation function. The ReLU function is expressed as follows:

(4) $$\begin{array}{c} G\left(X\right)=\max\left(0,X\right). \end{array}$$

ReLU function requires to meet the following conditions in the process of neural network training:

(5) $$\begin{array}{c} G\left(X\right)=\left\{\begin{array}{cc} 0, & x<0,\\ X, & x>0. \end{array}\right) \end{array}$$

The loss function shows the difference between the target area output by the model segmentation and the actual target area [17]. Since the area of NPC tumor and lymph node was relatively small in the MRI image, Dice loss was used as the model loss function. The Dice loss function mainly uses the Dice coefficient to evaluate the degree of overlap between the model output and the actual segmentation label. The calculation method of Dice coefficient is expressed as follows:

(6) $$\begin{array}{c} \text{Dice}=\frac{2\left|C\cap D\right|}{\left|C\right|+\left|D\right|}, \end{array}$$

where *C* is the predicted output map of the model and *D* is the gold standard drawn manually. Therefore, the range of the Dice coefficient is [0,1], and the closer the Dice coefficient is to 1, the better the model segmentation performance is. According to the Dice coefficient, the equation for calculating Dice loss is as follows:

(7) $$\begin{array}{c} \text{Loss}=1-\text{Dice}. \end{array}$$

The CNN-based intelligent segmentation model requires pixel standardization, horizontal flipping, and rotation to enhance the MRI image data for the acquired MRI images. The pixel standardization mainly adjusts the pixel range of the MR image of the three modalities of T1W, T2W, and T1C between [0,255]. The pixel conversion method can be expressed as

(8) $$\begin{array}{c} \hat{P}=\frac{P}{P_{\max}}\times 255. \end{array}$$

In equation (8), *P* is the pixel value of the original MRI image, *P*_max_ is the maximum pixel value on the entire dataset, and $\hat{P}$ is the pixel value of the MRI image after conversion. The *Z*-score algorithm is further used for standardization, and the calculation method is shown in equation (9), where *μ* and *δ* represent the mean and standard deviation of different modal images on each channel of the ImageNet data set, respectively, and *P* is the normalized pixel value:

(9) $$\begin{array}{c} P_{i}=\frac{\hat{P}-\mu }{\delta }. \end{array}$$

The three modal images of T1W, T2W, and T1C of NPC patients were made full use of. Multisequence cross convolutional (MSCC) method [18] was used for multisequence fusion to establish a 2D segmentation model, which was named 2D-ResUNet model. The 2D-ResUNet model had the advantages of small network parameters and fast fitting speed, but it cannot make full use of the topological structure information between medical imaging layers. Therefore, problems such as poor segmentation performance and discontinuous segmentation results between different layers can occur.

### 2.4. Multisequence Multidimensional Fusion Segmentation Model Based on CNN

The three-dimensional (3D) model can make full use of the interlayer information of the image to obtain continuous segmentation results, but its network parameters are huge. As a result, the fitting speed is slow, the training time is long, and even it is difficult to fit. Referring to the H-Dense UNet model proposed by Tran et al. [19], 2D and 3D were combined to establish a multidimensional fusion structure (MDFS) that combined the advantages of the 2D models to improve the segmentation performance of the model. Multidimensional fusion structure mainly includes multisequence 2D-ResUNet structure, 3D-ResUNet structure, and 2D + 3D fusion layer. It is assumed that the function *H* represents the process of transforming a 3D image into 2D one, and *H*^−1^ is the inverse process of the transformation. Then, the model input 3D image *I* is expressed as follows:

(10) $$\begin{array}{c} I_{2d}=H\left(I\right). \end{array}$$

In equation (10), *I* ∈ *R*^1×256×256×*b*×3^ and *b* is the image depth. Then, the three modal 2D images of T1W, T2W, and T1C are obtained as *I*_2*d*−T1W_, *I*_2*d*−T2W_, and *I*_2*d*−T1C_, respectively. It is assumed that the 2D network is *f*_2*d*_ and the 3D network is *f*_3  *d*_; then, the feature map *F*_2*d*_ and probability map *y*_2*d*_ of the multisequence 2D image after being processed by the multisequence 2D-ResUNet are expressed as follows:

(11) $$\begin{array}{c} F_{2d}=f_{2d}\left(I_{2d-\text{TW}},I_{2d-\text{T}2\text{W}},I_{2d-\text{T}1\text{C}};\theta_{2d}\right),\;F_{2d}\in R^{b\times 256\times 256\times 16}, \end{array}$$

(12) $$\begin{array}{c} y_{2d}=f_{2dc\;ls}\left(F_{2d};\theta_{2dc\;ls}\right),\;y_{2d}\in R^{256\times 256\times b\times 3}. \end{array}$$

In equations (11) and (12), *θ*_2*d*_ is the convolutional network parameter and *θ*_2*dc*  *ls*_ is the classification network parameter. To combine the results obtained from the 2D network with the 3D network, the feature map *F*_2*d*_ and probability map *y*_2*d*_ should undergo the following inverse transformation:

(13) $$\begin{array}{c} \tilde{F_{2d}}=H^{-1}\left(F_{2d}\right),\;\tilde{F_{2d}}\in R^{1\times 256\times 256\times 16\times b},\\ \tilde{y_{2d}}=H^{-1}\left(y_{2d}\right),\;\tilde{y_{2d}}\in R^{1\times 256\times 256\times 3\times b}. \end{array}$$

Further, $\tilde{y_{2d}}$ and *I* are merged and input into the 3D-ResUNet together; then the characteristic map of the 3D network is obtained as follows:

(14) $$\begin{array}{c} F_{3\;d}=f_{3d}\left(I,\tilde{y_{2d}};\theta_{3d}\right),\;F_{3d}\in R^{1\times 256\times 256\times 16\times b}. \end{array}$$

In equation (14), *θ*_3*d*_ is the 3D network parameter. After $\tilde{F_{2d}}$ and *F*_3*d*_ are summed, the 2D + 3D fusion layer and the classification layer are input to get the 3D segmentation result, which is as follows:

(15) $$\begin{array}{c} Y=\tilde{F_{2d}}+F_{3d},\\ H=f_{HF}\left(Y;\theta_{HF}\right),\\ y_{H}=f_{HFcls}\left(Y;\theta_{HFcls}\right). \end{array}$$

In equation (15), *θ*_*HF*_ is the network parameter of the convolutional layer *f*_*HF*_ of the fusion layer and *θ*_*HFcls*_ is the network parameter of the classification layer *f*_*HFcls*_.

The loss function of the multidimensional fusion structure model includes the loss of 2D-ResUNet and the loss of 2D + 3D fusion layer. The loss is expressed as equation (16), where *β* is the weight of 2D-ResUNet, *L*oss_2D_ is the 2D-ResUNet loss, and *L*oss_*F*_ is the 2D + 3D fusion layer loss:

(16) $$\begin{array}{c} \hat{\text{Loss}}=\beta \;{\text{Loss}}_{2\text{D}}+{\text{Loss}}_{F}. \end{array}$$

**Figure 2:**
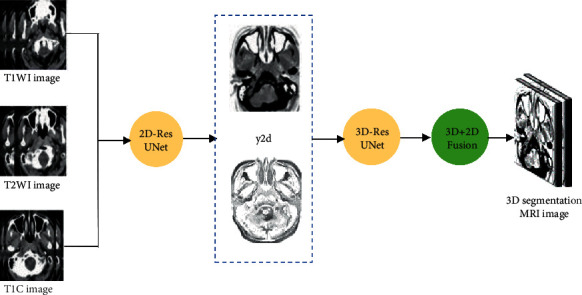
Operation flowchart of multisequence multidimensional fusion network model.

The multidimensional fusion structure model combines the advantages of fast 2D network fitting speed and full utilization of 3D network spatial information. The segmentation results of the 2D network guide the fitting of the 3D model, so as to quickly and effectively implement the training and testing of the model. The operation process of the multisequence multidimensional fusion network model is shown in Figure 2.

### 2.5. Evaluation of MRI Image Segmentation Performance

Dice coefficient, Hausdorff distance (HD), and percentage of area difference (PAD) were selected as the evaluation indexes of model segmentation effect. The calculation methods of HD and PAD are as follows:

(17) $$\begin{array}{c} \text{HD}\left(C,D\right)=\underset{a\in C}{\max}\left\{\underset{a\in D}{\max}\left[s\left(a,b\right)\right]\right\},\\ \text{PAD}=\frac{\left|C-D\right|}{C}. \end{array}$$

The Dice coefficient is often used to evaluate the similarity between the results of automatic image segmentation and the results of physicians' manual delineation [20], and its calculation method is as follows:

(18) $$\begin{array}{c} \text{Dice}\left(C,D\right)=\frac{2\left|C\cap D\right|}{\left|C\right|+\left|D\right|}. \end{array}$$

In the above equations, *s*(*a*, *b*) is the Euclidean distance between points *a* and *b*. The smaller the HD and PAD is, the closer the model segmentation result is to the manual delineation result and the better the performance is. *C* is the pixel set of the automatically segmented image, and *D* is the pixel set of the manually drawn image. The Dice value range is [0,1]; the larger the Dice value is, the closer the automatic segmentation result is to the manual delineation result.

### 2.6. Statistical Methods

SPSS 19.0 was employed for data statistics and analysis. Mean plus or minus standard deviation (*x* ± *s*) was how measurement data were expressed, and percentage (%) was how count data were expressed, which was tested by *χ*^2^ test. The difference was statistically considerable with *P* < 0.05.

## 3. Results

### 3.1. Analysis of MRI Image Tumor Lesion Segmentation Results Based on CNN Model

**Figure 3:**
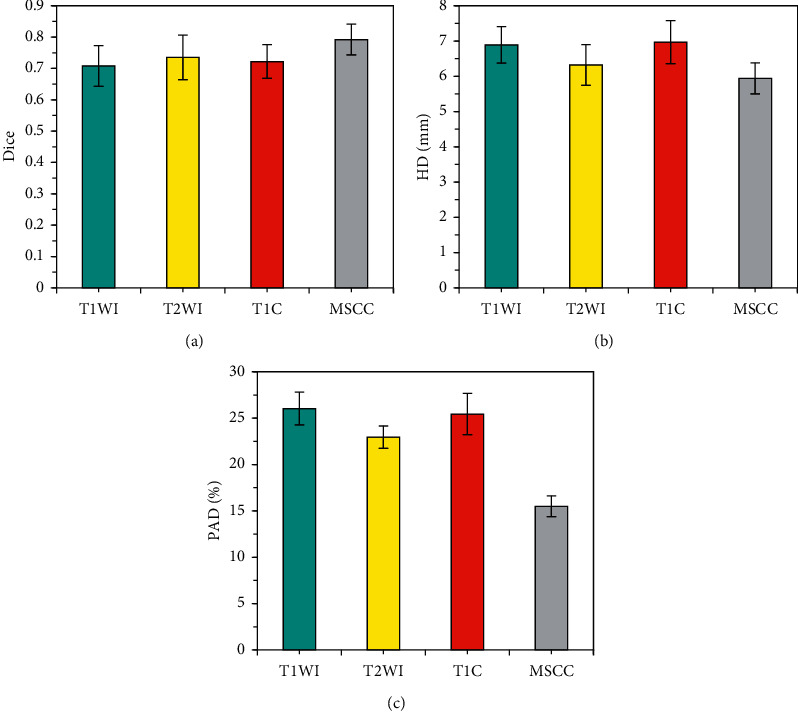
Analysis of segmentation results of tumor lesions in different MRI image blocks. (a) Comparison of Dice coefficients of tumor lesions in different MRI image blocks; (b) comparison of HDs of tumor lesions in different MRI image blocks; (c) comparison of PADs of tumor lesions in different MRI image blocks.

The doctor's outline of the lesion area was deemed as the gold standard, and the segmentation performance of the intelligent segmentation model based on CNN was evaluated. After all MRI images were amplified by the training set and divided into image blocks, three sequence image blocks of T1WI, T2WI, and T1C were obtained. The data of the independent test set was input and saved, the Dice coefficients, HD, and PAD of the three sequence image blocks and the MSCC method to process the image blocks were compared, and the results were shown in Figure 3. The maximum Dice coefficient of MSCC was 0.792 ± 0.045, and its HD and PAD were the smallest, which were 5.94 ± 0.41 mm and 15.96 ± 1.232%, respectively.

**Figure 4:**
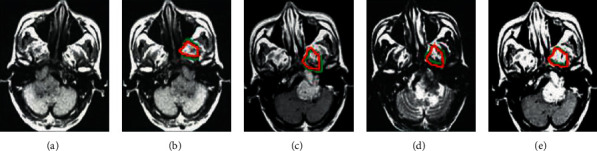
MRI tumor segmentation results of a 42-year-old male with NPC lesions invading the brainstem. (a) Input MRI image; (b) T1WI image; (c) T2WI image; (d) T1C image; (e) MSCC processed image.

CNN model was employed to segment tumor lesions in different MRI images (Figure 4). The green was the tumor focus area marked by the doctor, and the red was the tumor focus area automatically marked by the CNN model in the study. After MSCC processing, the marked area of the tumor lesion in the image became closer to the area manually outlined by the doctor.

### 3.2. Segmentation Results of Lymph Node Lesions in MRI Images Based on CNN Model

**Figure 5:**
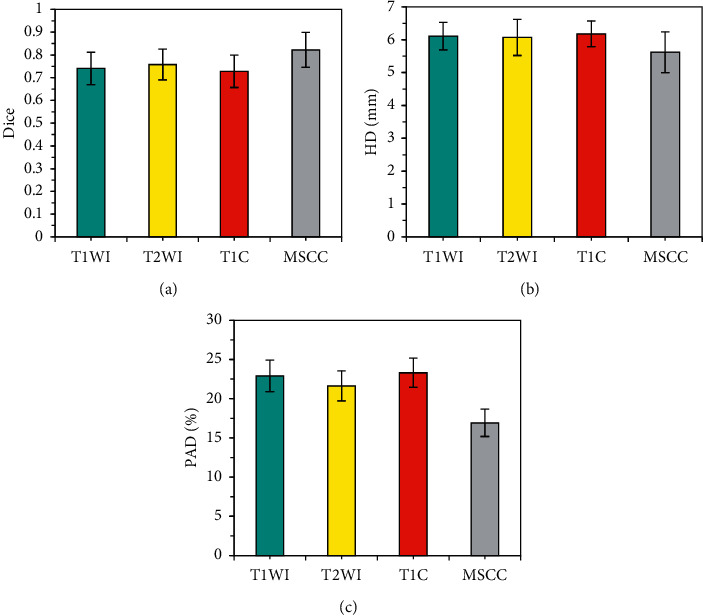
Segmentation results of lymph node lesions in different MRI images. (a) Comparison of Dice coefficient of lymph node lesion segmentation in different MRI images; (b) comparison of HD of lymph node lesion segmentation in different MRI images; (c) comparison of PAD of lymph node lesion segmentation in different MRI images.

The segmentation of the lymph node lesions of NPC patients was analyzed (Figure 5), the Dice coefficient of MSCC was the largest (0.822 ± 0.077), and its HD and PAD were the smallest, which were 5.62 ± 0.62 mm and 16.92 ± 1.74%, respectively.

**Figure 6:**
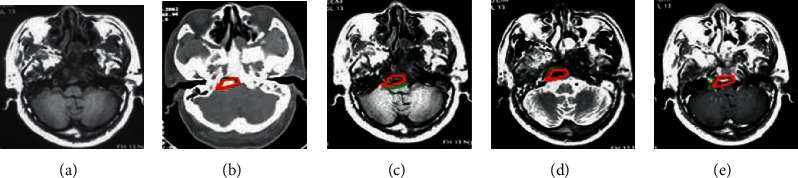
MRI lesion segmentation results of NPC lymph node metastasis (male, 37 years old). (a) Input MRI image; (b) T1WI image; (c) T2WI image; (d) T1C image; (e) MSCC processed image.

CNN model was utilized to segment lymph node lesions in different MRI images (Figure 6). Green was the lymph node area marked by the doctor, and red was the lymph node lesion area automatically marked by the CNN model in this study. After MSCC processing, the marked area of the lymph node lesion in the image became closer to the area manually delineated by the doctor.

### 3.3. Convergence Performance of MSCC-MDF

**Figure 7:**
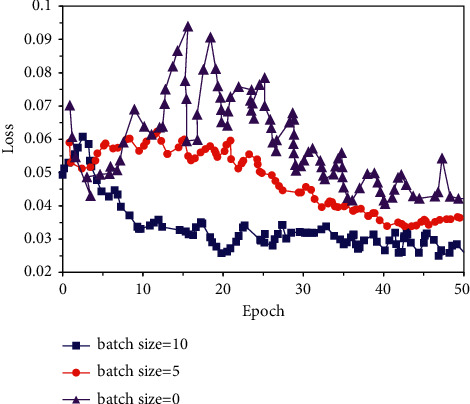
Convergence curve of MSCC-MDF.

The convergence performance of the MSCC-MDF under different batch size conditions was analyzed, and the results were shown in Figure 7. With the continuous increase of batch size, the convergence speed of the MSCC-MDF increased significantly. When batch size = 10, the convergence speed was the fastest, starting from the 8th epoch. When batch size = 0, the convergence curve fluctuated significantly within 50 epochs; especially, severe oscillations occurred in the first 30 epochs. When batch size = 5, the convergence curve was relatively gentle, and the convergence speed was moderate. Moreover, the convergence curve did not experience serious fluctuations in the loss value within 50 epochs. Therefore, the batch size was set to 5.

### 3.4. Tumor Lesion Segmentation Results by MSCC-MDF

**Figure 8:**
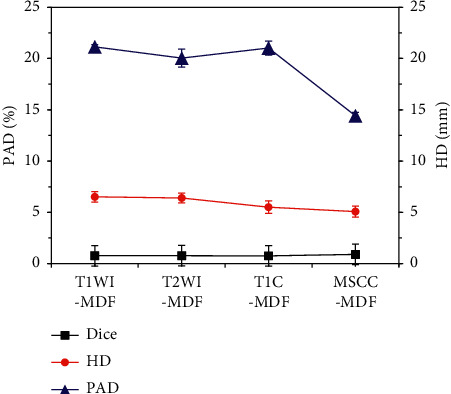
NPC tumor lesion segmentation results by MSCC-MDF.

The multisequence multidimensional fusion segmentation model was used to segment tumor lesions in NPC patients (Figure 8). The Dice coefficient of MSCC-MDF was the largest (0.896 ± 0.09), and the HD and PAD were the smallest, which were 5.07 ± 0.54 mm and 14.41 ± 1.33%, respectively.

**Figure 9:**
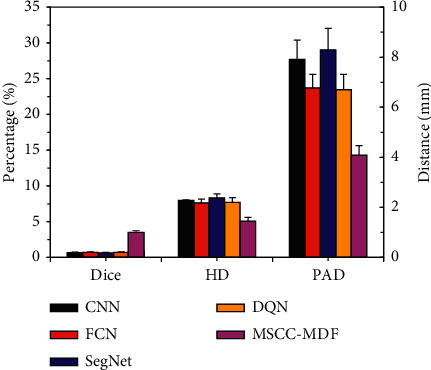
Comparison of NPC tumor lesion segmentation results among different algorithms. ^*∗*^Considerable difference compared with other algorithms (*P* < 0.05).

The segmentation results of the MSCC-MDF established in this research were compared with those of CNN [21], fully CNN (FCN) [22], SegNet [23], and deep Q-network (DQN) [24] (Figure 9). The Dice coefficient of the MSCC-MDF model was lower than other algorithms, and the difference was considerable (*P* < 0.05). HD and PAD of the MSCC-MDF model were both higher than other algorithms (*P* < 0.05).

## 4. Discussion

In this research, an intelligent segmentation model of MRI images was established based on the CNN algorithm and applied to the diagnosis of NPC. The results showed that the Dice coefficient of the MSCC processed MRI image segmented by CNN was the largest (0.792 ± 0.045), and the HD and PAD were the smallest, which were 5.94 ± 0.41 mm and 15.96 ± 1.232%, respectively. These results showed that, with MRI images of different sequence as the multichannel input of the model, the diversity of different sequence features gradually decreased after convolution and pooling. The segmentation performance of MSCC processed MRI images was improved. MSCC separately fused the feature maps of the same resolution in different sequences and input them into the decoder, so that the information of different sequences was preserved to the greatest extent, thus increasing the segmentation performance of the model [25]. Dal-Bianco et al. [26] pointed out that the effective fusion of multisequence MRI images can improve the segmentation performance of the model, which was consistent with this research. The training and fitting speed of the 2D model was fast, but it lost the interlayer structure relationship in the 3D space [27], so its segmentation effect was not continuous and required to be further improved.

Based on the 2D model, an MSCC-MDF was further constructed and applied to the diagnosis of NPC. The results showed that the Dice coefficient of the MSCC-MDF was the largest (0.896 ± 0.09), and its HD and PAD were the smallest, which were 5.07 ± 0.54 mm and 14.41 ± 1.33%, respectively. The Dice coefficient of the MSCC-MDF model was lower than other algorithms (*P* < 0.05), and both HD and PAD were higher than other algorithms (*P* < 0.05). These results showed that the MSCC-MDF had good performance in segmentation of MRI images. The segmentation evaluation indexes of the MSCC-MDF model were significantly better than the 2D model, which showed that the performance of multisequence fusion was better than single-sequence segmentation. Men et al. [28] used FCN with an encoding-decoding structure to segment tumor lesions and lymph node lesions in NPC patients and obtained Dice values of 0.81 and 0.62, respectively. The Dice value of the MSCC-MDF model was significantly improved. The MSCC-MDF model made full use of the interlayer information of MRI images to improve the continuity of MRI image segmentation. Moreover, it combined the advantages of 2D and 3D models, and it significantly improved the segmentation performance of the model while increasing the fitting rate.

## 5. Conclusion

In this research, an intelligent segmentation model 2D-ResUNe for MRI images was established based on the CNN algorithm. Further fusion of 2D and 3D models was implemented to establish an MSCC-MDF segmentation model, which was applied to the diagnosis of NPC patients. The results showed that the MSCC-MDF model had ideal performance in segmentation of MRI images of NPC patients. However, there are still some shortcomings in this research. The sample size of this research is small, and it is necessary to increase the sample size for further verification in future work. In summary, the MSCC-MDF model significantly improves the segmentation performance of MRI images of NPC patients, which provides a reference for the diagnosis of NPC.

## Data Availability

The data used to support the findings of this study are available from the corresponding author upon request.
